# Defining a metabolic landscape of tumours: genome meets metabolism

**DOI:** 10.1038/s41416-019-0663-7

**Published:** 2019-12-10

**Authors:** Chandan Seth Nanda, Sharavan Vishaan Venkateswaran, Neill Patani, Mariia Yuneva

**Affiliations:** The Francis Crick Institute, 1 Midland Road, London, UK

**Keywords:** Cancer metabolism, Cancer genomics

## Abstract

Cancer is a complex disease of multiple alterations occuring at the epigenomic, genomic, transcriptomic, proteomic and/or metabolic levels. The contribution of genetic mutations in cancer initiation, progression and evolution is well understood. However, although metabolic changes in cancer have long been acknowledged and considered a plausible therapeutic target, the crosstalk between genetic and metabolic alterations throughout cancer types is not clearly defined. In this review, we summarise the present understanding of the interactions between genetic drivers of cellular transformation and cancer-associated metabolic changes, and how these interactions contribute to metabolic heterogeneity of tumours. We discuss the essential question of whether changes in metabolism are a cause or a consequence in the formation of cancer. We highlight two modes of how metabolism contributes to tumour formation. One is when metabolic reprogramming occurs downstream of oncogenic mutations in signalling pathways and supports tumorigenesis. The other is where metabolic reprogramming initiates transformation being either downstream of mutations in oncometabolite genes or induced by chronic wounding, inflammation, oxygen stress or metabolic diseases. Finally, we focus on the factors that can contribute to metabolic heterogeneity in tumours, including genetic heterogeneity, immunomodulatory factors and tissue architecture. We believe that an in-depth understanding of cancer metabolic reprogramming, and the role of metabolic dysregulation in tumour initiation and progression, can help identify cellular vulnerabilities that can be exploited for therapeutic use.

## Background

Altered metabolism is a hallmark of cancer, and metabolic reprogramming in cancer cells is observed in the major pathways of central carbon metabolism. Exploiting such global metabolic alterations therapeutically can be challenging due to the essential nature of these pathways in both normal and cancer cells. Choosing metabolic interventions based on genomic and metabolic data could lay the foundation for the successful use of metabolism-based anti-tumour strategies. A recent example of this is enasidenib, an isocitrate dehydrogenase 2 (IDH2) inhibitor, which has been approved by the Food and Drug Administration (FDA) for the treatment of relapsed or refractory acute myeloid leukaemia (AML) in patients bearing *IDH2* mutations.

Metabolic reprogramming can be a result of either mutations in metabolic genes themselves or oncogenic mutations in signalling pathways. The latter can result in altered metabolic gene expression or differential post-translational modifications of metabolic enzymes that can lead to differential activity or protein localisation. In addition, metabolic reprogramming in cancer cells can also be due to a metabolic imbalance in native tissue homoeostasis, such as in the case of hypoxic or inflamed tissues, or metabolic disease conditions including obesity and diabetes. In such cases, metabolic imbalance may precede genetic changes and lead to tumour initiation. Cells bearing oncogenic mutations have the inherent advantage of self-propagation by expansion. Lineage tracing of these inheritable genetic lesions has shown that tumours often progress by neutral or branched clonal expansion. Therefore, tumour-initiating mutations should represent the tumour bulk and targeting cells that harbour these mutations could render a therapeutic advantage on the entire tumour. Therefore, understanding the relationship between genetic and metabolic changes, as well as the role of these interactions in tumour initiation, is essential for designing efficient therapeutic approaches targeting the metabolism of tumours.

A viable option is to identify metabolic vulnerabilities of cancer cells with well-documented oncogenic alterations. However, even though some synthetic lethal pairs might appear perfect for abolishing an entire tumour cell population, they might not work efficiently in the context of tumour in a patient. This can be due to the influence of multiple factors that contribute to inter- and intra-tumour metabolic heterogeneity during tumour development and progression. The factors affecting the metabolic state of a cancer cell can be intrinsic, including aberrant oncogenic signals and/or metabolic pathways and/or extrinsic, including immune cells, cancer-associated fibroblasts (CAFs) and stroma, as well as oxygen and nutrient gradients. These factors must be taken into consideration before defining a line of treatment based on the analysis of clinical samples. Therefore, an anti-cancer therapy aimed at metabolic pathways in tumours might be successful by integrating multimodal data (transcriptomic, genomic and metabolic data) in conjugation with information about factors affecting the tumour microenvironment (TME). Apart from therapy, this information could provide valuable insights into metabolic markers for tumour initiation, progression and metastasis.

## Pan-cancer metabolic gene expression data highlight metabolic reprogramming patterns across tissues

Genome instability, mutations and metabolic reprogramming are hallmarks of cancer. However, little is known about how each of these influence one another. One way to delineate this is by understanding the transcriptional outcome. Uniquely positioned, the transcriptional output is directly downstream of genomic instability, mutations and upstream of metabolic pathways. Although transcriptomic data can provide useful insights into metabolic gene expression, it does not reflect metabolic regulation or directionality of metabolic flux. Keeping these limitations of transcriptomic data in mind, it is still possible to identify valuable information about the dysregulation of metabolic pathways in different types of tumours and infer their relationship with tumour-driver mutations.

The availability of large pan-cancer genomic and transcriptomic annotated datasets permits ready comparisons of native tissues and cancer samples. They provide a global view of metabolic remodelling throughout different cancers, as well as how they diverge from native tissue.^1–4^ RNA-sequencing data from The Cancer Genome Atlas across 26 cancer types and normal tissues, and microarray data from 22 primary tumour types and the corresponding normal tissues have revealed differential expression of metabolic genes, confirming a global role of altered metabolism in cellular transformation (see Table 1). Importantly, while metabolic expression patterns differ significantly between normal tissues, they are less marked between tumours from different sites, suggesting some commonality of cancer metabolism. However, strong similarities between tumours and their native tissues also provide evidence for some tissue-specific metabolic hard wiring.^2,4^ This analysis demonstrates that glycolysis is among either the most dysregulated^1^ or frequently and significantly upregulated^2,4^ pathways in most cancer types (see Table 1). These results are consistent with the increased glucose uptake observed in many tumours, which is extensively used for tumour diagnostics by positron emission tomography (PET) scans.^5^ Increased glucose catabolism through glycolysis into lactate even under normal oxygen conditions, termed aerobic glycolysis, has been considered as a general phenomenon of tumours, stemming from the initial observation by Otto Warburg in 1927. However, it is also observed in normal proliferating cells.^6^ Aerobic glycolysis yields less ATP than its full oxidation through oxidative phosphorylation (OxPhos), providing the subject for a long-time discussion about its role in proliferating cells. Metabolic modelling and calculating energy and biosynthetic demands of proliferating cells suggest that when cells need both energy and biomass produced at a fast rate, aerobic glycolysis serves to overcome the limited capacity of mitochondrial OxPhos to oxidise NADH, and uncouples the production of NADH from biosynthetic processes.^7^ Pan-cancer studies also consistently demonstrate that together with glycolysis, there are other pathways for macromolecular biosynthesis and biomass build-up, which are frequently and significantly upregulated in many of the cancer samples in comparison with normal tissues, such as purine and pyrimidine biosynthesis^2,4^ and aminoacyl-t-RNA biosynthesis (see Table 1). Interestingly, the expression of the Krebs cycle and OxPhos genes demonstrates heterogenous behaviour between cancer types,^2,4^ suggesting that the role of OxPhos is not universal across different cancers and can be determined by the environmental cues or tissue-specific functions of genetic and signalling drivers, which are discussed further in this review.

**Table 1:**
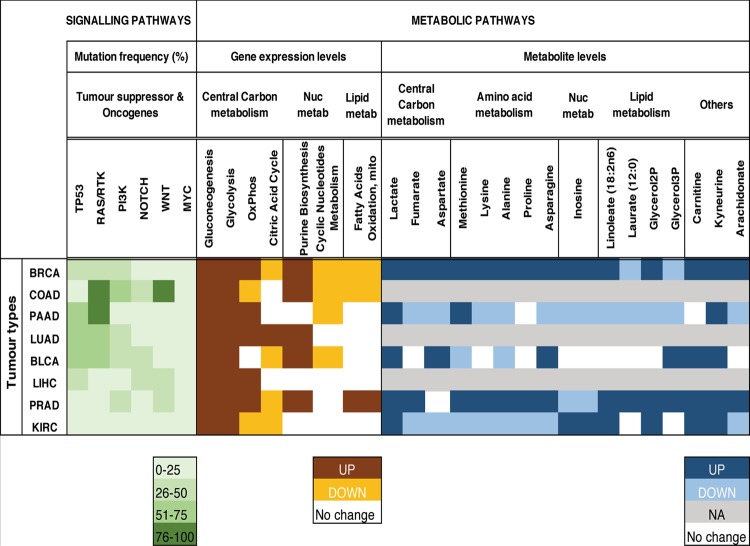
Patterns of genomic, transcriptomic and metabolic changes in different human tumours.

The heatmap coloured in various shades of green shows the percentage of gene mutational frequencies in different cancers (data obtained from TCGA study by Sanchez-Vega et al. 2018). The heatmap coloured in various shades of ochre shows the differential metabolic gene expression levels in different cancers (data obtained by Gaude and Frezza 2018, Rosario et al., 2018, Hu et al., 2013). The heatmap coloured in various shades of blue shows the differential metabolite levels by mass spectrometry in different cancers (data obtained by Reznik et al., 2018).

BRCA breast invasive carcinoma, COAD colon adenocarcinoma, PAAD pancreatic adenocarcinoma, LUAD lung adenocarcinoma, BLCA bladder urothelial carcinoma, LIHC liver hepatocellular carcinoma, PRAD prostate adenocarcinoma, KIRC kidney renal clear cell carcinoma

While pan-cancer transcriptomic analysis can provide a valuable global view of metabolic reprogramming in different cancer types, applying transcriptomic changes to metabolic phenomena can be problematical. Some of the challenges include stoichiometric and kinetic assumptions, properties of different metabolic enzyme isoforms and inherent limitations regarding post-transcriptional, post-translational and/or epigenetic regulation. Some studies have combined transcriptomics analysis with evaluating the level of metabolites.^8–10^ In many cases, the levels of specific metabolites correlated with the expression of metabolic genes, supporting a conclusion about the change in the activity of a pathway. For example, integrating the transcriptomic and metabolomic data from Teranuma et al.^8,11^ demonstrated a positive correlation of glycolytic genes and lactate levels, and the expression of nucleotide and DNA synthesis genes with the levels of nucleotides, confirming the increased activity of glycolysis and nucleotide biosynthesis in breast cancers.^4^ A similar correlation was observed in colon cancer samples.^12^ Furthermore, combining metabolomics data from several pan-cancer studies has confirmed the accumulation of lactate and demonstrated the increased levels of acyl-carnitine metabolites in tumours across seven tissue types (in different studies; see Table 1). On the other hand, the lack of linearity and discordance between transcriptomic and metabolomic changes in renal cancer has, for example, prompted the development of an analytical pipeline and visualisation tool (metabologram) to bridge the gap.^13^

Although these studies may suffice to provide a global view of the metabolic changes throughout cancer types and within specific subtypes, they do not provide evidence for a directionality of metabolic fluxes and could miss essential information about some specific metabolic changes. Although there are efforts to obtain information about metabolic fluxes from metabolite concentrations,^11,14–16^ it is still a challenge. To learn about the activity and directionality of a specific pathway, as well as a contribution of specific nutrients that fuel them, the use of stable isotope labelling is required. Although this approach is now routinely being used in in vitro and in vivo preclinical models, human cancer studies using stable isotope labelling are limited.^17–20^ For example, using ^13^C-labelled glucose in patients with non-small-cell lung carcinomas (NSCLCs) and tumour tissue slices, Sellers et al.^20^ confirmed the increased activity of glucose catabolism through glycolysis and into nucleotide and serine biosynthesis in squamous cell carcinomas (SCCs), a subtype of NSCLCs, which was also identified based on the level of metabolic enzyme genes. However, the authors demonstrated further the increased utilisation of glutamine in SCCs through a reductive direction of the Krebs cycle, which was impossible to infer from the gene expression pattern alone.^20^ In another state-of-the-art study, analysis of the ^13^C-glucose-derived pattern in lactate and the Krebs cycle intermediates in NSCLCs identified that carbon sources other than glucose significantly contribute to activity of the Krebs cycle in some tumours.^18^

**Fig. 1:**
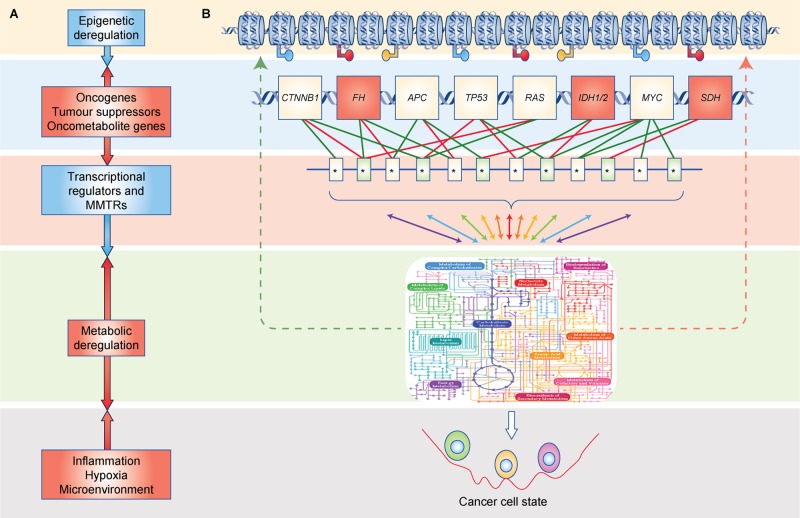
Schematic diagram of the metabolic nodes of tumour initiation and their directional regulation in defining a cancer cell state and metabolic landscape in cancer. **a** Genetic alterations in oncogenes, tumour suppressors and oncometabolite genes can lead to metabolic reprogramming. Alternatively, metabolic dysregulation can be an initiator of cellular transformation. Tumour-initiating nodes in the cancer cell regulatory network are highlighted in red. **b** Visual depiction of the regulatory network defining the cancer cell state. Oncometabolite genes are highlighted in orange boxes.

Together with providing a global picture on metabolic remodelling, pan-cancer transcriptomic analysis has already provided information about the regulation of metabolic processes in different tumours. An important discovery from large-scale pan-cancer studies is of master metabolic transcriptional regulators (MMTRs) (see Fig. 1). MMTRs have been shown to comprise transcription factors and microRNAs across different cancer types^3^ and orchestrate functional gene sets with convergent pathway-level effects, resulting in stable metabolic subtypes.^1,3^ Shared MMTRs between processes, such as nucleotide metabolism, amino acid metabolism and the Krebs cycle, may help to co-ordinate metabolic crosstalk between pathways,^3^ indicating converging polygenic information into a defined metabolic signature.^21^ Hence, the attempts to dissect metabolic programmes in a pan-cancer setting have most importantly helped to identify metabolic commonalities across cancer types and the existence of these MMTRs.

Taken together, from pan-cancer studies we can start to understand not just the extent of changes in metabolic gene expression in different cancers, but also the regulation that occurs at the transcriptomic level. However, to be able to design efficient therapeutic approaches, the connection should be made between genomic and metabolic changes. In the sections below, we provide details of the genomic master players that work in conjunction with MMTRs to drive metabolic reprogramming associated with tumorigenesis.

## Metabolic reprogramming occurs downstream of oncogenic signalling pathways

In normal cells, different signalling pathways, such as WNT signalling, TP53, RAS and phosphoinositide 3-kinase (PI3K) signalling pathways control the cell cycle, growth, survival and cell fate in accordance with the availability of nutrients and oxygen (reviewed in refs. ^22,23^). The nutrient-sensing response (such as the sestrin–mammalian target of rapamycin [mTOR]–AMP-activated protein kinase [AMPK] axis) to oxidative and xenobiotic stress through Keap1–Cul3 ubiquitin E3 ligase^24^ also occurs via signalling pathways. Importantly, most of these signalling cascades relay signals into the nucleus via transcriptional factors or cofactors to regulate the expression of genes encoding metabolic enzymes or the activity of metabolic enzymes themselves through post-translational modifications (reviewed in refs. ^25,26^). For example, regulation of metabolic gene transcription has been shown by *MYC* (reviewed in ref. ^27^), *RAS* (reviewed in ref. ^28^) and *TP53* (reviewed in ref. ^29^).

In cancer cells, dysregulation of the components of these signalling pathways, including loss- or gain-of-function mutations, amplifications and deletions, leads to uncontrolled proliferation (such as *TP53, APC, KRAS, MYC* and *PI3K*) and an imbalance in the redox state (such as *NRF2, KEAP1*; reviewed in refs. ^22,25,27,28,30–32^). In recent years, many studies employing in vitro and in vivo models have associated the activity of metabolic pathways with the activation of specific oncogenes and loss of tumour suppressors. Even though it is challenging to do in heterogeneous patient samples, this has been attempted in a few pan-cancer studies. Hu et al. demonstrated that glycolysis strongly correlates with the expression of *RAS* and genes from the mitogen-activated protein kinase (MAPK) pathway.^2,33^ They also identified that CDC42 expression correlates with glycolysis, nucleotide and amino acid biosynthesis, supporting the role of these pathways in maintaining the proliferation of cancer cells. Peng et al. explored the relationship between either mutation driver genes or drivers of somatic copy-number alterations and metabolic expression subtypes, and demonstrated heterogeneous patterns between cancer types.^3,34^

Furthermore, several studies have focused on specific cancer types identifying the correlation between different genetic drivers and the expression of metabolic genes and pathways. In breast cancer, *TP53* mutations^35^ and *BRCA* mutations^36^ were associated with an upregulated glycolysis gene signature, and *HER2* gene amplifications with upregulated glycolysis and choline metabolism.^37^ In lung squamous carcinomas, increased Notch signalling was associated with the increased expression of glycolysis, the Krebs cycle, nucleotide metabolism and one-carbon metabolism genes.^20^

It is well established that tumours are not a collection of uniform cells, but instead have clonal populations driven by different genetic lesions. These distinct clonal lineages generate genetic heterogeneity within a tumour. Given this genetic heterogeneity, a clear picture of how genetic drivers influence metabolic programmes dictated by one another would help to predict a patient’s outcome. However, there are only a few in vivo studies evaluating the metabolic interaction between different genetic lesions. *MYC* is one of the most frequently altered genes in cancer and a transcriptional factor regulating metabolism at multiple levels (reviewed in ref. ^38^). Using mouse models of lung tumorigenesis, we have recently demonstrated that *NOTCH1* can amplify the metabolic programme implemented by *MYC*.^20^ *MYC* and *HER2* were also shown to co-operate in increasing the expression of lipoprotein lipase (LPL) and cholesterol biosynthesis genes.^39^ Importantly, copy-number variation can also affect the metabolic profile and hence cell fate. The gain of more copies of mutant KRAS can transform the metabolic profile of lung cancer cells from a more glycolytic state to an enhanced Krebs cycle and glutathione-synthetic state, which enhances tumour-cell invasiveness.^40^

Despite these elegant attempts to delineate the effect of genetic mutations in oncogenic signalling pathway components to metabolism, many questions still remain unanswered. Importantly, it remains an outstanding question as to how combinations of driver lesions change individual metabolic pathways and the entire metabolic landscape of cancer cells from different tissue types. It is essential to address this question in order to identify metabolic vulnerabilities of cells with a specific genomic makeup in the context of genetically heterogeneous tumours.

## Metabolic reprogramming can act as a driver of cellular transformation

As discussed above, it is now clear that metabolic reprogramming is an inherent hallmark of cancer and occurs in tumours driven by different signalling pathways among different tissues. However, it is pertinent to ask whether metabolic reprogramming is a cause or a consequence of the transformation process. In the following section, we focus on metabolism as an initiator of cancer through genetic and non-genetic alterations (see Fig. 1).

### Genomic mutations in metabolic pathways as a driver of tumorigenesis

Although Otto Warburg proposed that cancer is a disease of dysfunctional mitochondria back in 1956, this hypothesis has been scrutinised over the subsequent years.^41^ Genetic mutations in key mitochondrial enzymes of the Krebs cycle, such as succinate dehydrogenase (*SDH*), fumarate hydratase (*FH*) and *IDH1/2*, have recently been identified as cancer initiators. Metabolites accumulating as a result of these mutations, succinate, fumarate and d-2-hydroxyglutarate (D-2HG) respectively, are termed oncometabolites. Of these, D-2HG is produced from α-ketoglutarate (KG) as the result of a neomorphic enzymatic activity of the dimer formed between mutant and wild-type IDH subunits.^42^ Mutations in *SDH*, *IDH1/2* and *FH* are associated with different tumour types. Tumours carrying loss-of-function mutations in *SDH* include pheochromocytomas, paragangliomas, renal cancers, gastrointestinal cancers and some leukaemias, loss-of-function mutations in *FDH* are observed in infantile encephalopathy and renal cancer and gain-of-function mutations in *IDH1/2* are found in colon carcinomas, gliomas, glioblastomas, chondrosarcomas, cholangiocarcinomas and AMLs (reviewed in ref. ^43^).

All three oncometabolites act as inhibitors of KG-dependent dioxygenases, including the Jumonji-C-domain-containing histone lysine demethylases (KDMs) and the ten–eleven translocation (TET) family of 5-methylcytosine (5mC) hydroxylases, which results in epigenetic alterations that affect the expression of genes involved in cell differentiation and consequently induces transformation (reviewed in ref. ^44^) (see Fig. 1). Both succinate and fumarate can allosterically inhibit α-ketoglutarate-dependent prolyl-hydroxylase (PHD) and thus stabilise hypoxia-inducible factor 1α (HIF1α), which creates pseudohypoxia.^45^ The mechanistic insights of HIF activation during hypoxia will be detailed later in this review. Although HIF1α accumulation and upregulation of its target genes has been shown in *IDH1*-mutant cells,^46^ the relationship between D-2HG and HIF1α seems to be context-dependent (reviewed in refs. ^47,48^). D-2HG production consumes NADPH and competes with other NADPH-requiring pathways required for cellular survival, which makes *IDH1*-mutant cells more sensitive to oxidative stress.^49^ Accumulation of D-2HG can also directly inhibit ATP synthase^50^ and the α-ketoglutarate-dependent dioxygenase alkB homolog (ALKBH) DNA-repair enzyme that leads to DNA damage.^51^ Although D-2HG can be produced in the presence of *IDH1/2* mutations, its enantiomer L-2HG is produced under hypoxic conditions by lactate dehydrogenase A (LDHA) and malate dehydrogenase 1/2 (MDH1/2), but does not exert the same effect.^52–54^ In addition, ectopic MYC overexpression has been shown to increase D-2HG production through increasing glutamine catabolism in mammary epithelial cells, and an increased MYC transcriptional signature is associated with higher levels of D-2HG.^8^

Each of the oncometabolites and enzymatic reactions that produce them also have specific effects that can contribute to cellular transformation. Fumarate can irreversibly modify the cysteine residues in Kelch-like ECH-associated protein 1 (KEAP1) by succination, thereby activating nuclear factor erythroid-2-related factor 2 (NRF2) and promoting a reductive environment supporting cellular proliferation.^55^ In addition, analysis on a pan-cancer cell line set (from Cancer Cell Line Encyclopedia [CCLE]) has highlighted that mutations in the KEAP1 gene are associated with higher levels of redox metabolites such as glutathione (GSH/GSSG) and NADP^+^.^56,57^

Fumarate can also cause succination and inhibition of aconitase,^58^ and FH mutations can lead to auxotrophy of arginine via deregulation of the urea cycle and purine biosynthesis pathways.^59^

In this review, we have mainly focused on metabolic genes that initiate cancer; however, there are several metabolic genes that are mutated during cancer progression. For instance, mutations or copy-number variation in genes such as phosphoglycerate dehydrogenase (PHGDH) have also been linked to promoting breast cancer.^60,61^

These studies highlighted above demonstrate the role of metabolic pathways in supporting tumorigenesis induced by oncogenes and tumour suppressors. In the following sections, we detail the non-genetic ways in which metabolic pathways can be altered and contribute to tumorigenesis.

### Non-genetic changes in metabolism leading to tumorigenesis

Although the contribution of genetic factors in cancer is clear, the contributions of non-genetic metabolic alterations is still under assessment. Several studies have highlighted extensive metabolic reprogramming in the TME by hypoxia, nutrient availability and inflammation in cancer. Although these factors play regulatory roles in cancer, by modifying the TME to favour tumour growth, there is evidence that they can also act as tumour initiators in some cases. In this section, we will highlight a few scenarios that indicate the possibility of these factors in causing cancer.

#### Metabolic dysregulation diseases

Diseases such as diabetes and obesity exemplify how metabolic dysregulation can be complex and triggers multiple simultaneous changes in tissue microenvironments, such as oxidative stress and inflammation and cellular signalling changes, including the activation of the PI3K or AMPK signalling pathways. Although the association of diabetes with cancer is not new, a recent meta-analysis with over 20 million individuals has established a highly significant relationship between diabetes and cancer.^62^ Furthermore, several studies using preclinical models have demonstrated the role of insulin signalling and diabetes in promoting tumorigenesis.^63–66^ Obesity has been linked to high levels of circulatory leptin, but low levels of adiponectin. Leptin has body-wide metabolic effects, including activation of the PI3K–AKT–mTOR pathway and hence increased cell proliferation.^67^ Lack of adiponectin, on the other hand, leads to reduced activation of the nutrient sensor AMPK, which leads to uncontrolled proliferation.^68^ Moreover, it has been shown that secretion of leptin and adiponectin can lead to insulin resistance, which impairs the insulin sensitivity of the tissue.^69^ Adipocytes of obese patients produce monocyte chemoattractant protein-1 (MCP-1), which leads to an enhanced influx of monocytes and production of cytokines, and hence initiation of inflammation (reviewed in ref. ^70^). Chronic inflammation in adipocytes leads to their enhanced lipolysis, which produces excessive saturated free fatty acids. These free fatty acids, in turn, activate Toll-like receptor 4 (TLR4)-based nuclear factor κB (NF-κB) signalling^71^ and the reactive oxygen species (ROS) pathway^72^ to drive cell growth, proliferation and migration of, for instance, breast cancer cells.

#### Inflammation

It is clear that inflammation is a hallmark of diseased tissues, such as in the case of diabetes, obesity and other metabolic diseases. The role of inflammation in tumorigenesis has long been a focus, and multiple studies have established a direct association between enhanced inflammation and tumorigenesis. Many gastrointestinal cancers are an outcome of chronic inflammation,^73^ including colorectal cancer, hepatocellular carcinoma, pancreatic ductal adenocarcinoma and gallbladder cancer (reviewed in refs. ^73,74^). In all of these cases, infections, wounding or environmental toxic exposures result in metabolic perturbations that precede genomic instability and accumulation of driver mutations.

Inflammation preceding tumorigenesis can be a compounded scenario of increased ROS and hypoxia, both of which can cause DNA damage and lead to genomic instability.^70^ For example, in the case of persistent infections, the production of ROS and reactive nitrogen species (RNS) by leucocytes and other phagocytic cells leads to DNA damage in leucocytes as well as epithelial cells. These ROS/RNS not only damage the DNA and cause genomic instability, but also lead to protein carbonylation, which is an irreversible protein modification. These findings establish that there is a direct influence of inflammation-associated metabolic processes on tumour initiation. These correlations can be strengthened by the observation that there are deregulated networks that are observed throughout different cancer types irrespective of the type of genetic mutations. An example of this is the conserved araX network, which is the arachidonate and xenobiotics pathway network.^75^ Of note, perturbations in arachidonic acid metabolism have been linked to immunomodulatory factors, which produce mitogens that can alter cancer cell growth.^76,77^ To add to this, xenobiotic pathways contribute to redox imbalance, promoting genotoxicity, which can definitely contribute to cancer initiation.^78^

#### Hypoxia

Hypoxia is a feature of cancer, and it is clear from most metabolic and genomic studies that HIFs are activated in many cancers. The hypoxic environment and HIF1α stabilisation have been shown to be required for AKT-mediated transformation of melanocytes.^79^ Similarly, the HIF1α pathway has been shown to activate insulin growth factor signalling and platelet-derived growth factor (PDGF) receptor (PDGFR) signalling,^80,81^ leading to increased self-renewal and differentiation in vitro. Moreover, HIF1α signalling has been shown to induce telomerase activity through human telomerase reverse transcriptase (hTERT) expression, which in turn leads to cellular transformation.^82^ In addition to this, the HIF1 complex initiates transcription of metabolic genes, survival and growth factors (such as PDGFB, transforming growth factor β [TGFβ], insulin-like growth factor-II [IGF-II]) and angiogenesis factors, including vascular endothelial growth factor [VEGF] (reviewed in ref. ^83^) to initiate cancer. These in vitro studies suggest the role of a HIF1-dependent hypoxic response in enhancing gene expression of oncogenic components that promote cellular transformation.

Taken together, the findings mentioned above show how tumours can be initiated in metabolically unstable or perturbed environments, such as in the case of hypoxia, acidosis and inflammation. Systematic studies on the stepwise progression of tumours will be able to help sketch the molecular portraits and identify mutational events and metabolic switches. Imaging techniques based on mass spectrometry and cytometry (discussed later in this review) will also be able to help identify metabolites that precede hyperplasia and hence tumour development.

## Levels and contributors of metabolic heterogeneity

The metabolic programme of cancer cells can be modified over the entire course of tumour development. So far, we have described the intrinsic factors, including alterations in oncogenic signalling and metabolic pathways, that result in metabolic reprogramming. Apart from these factors, there are several other extrinsic factors that act as regulators of the TME, support tumour progression and shape tumour metabolism. The combination and interaction of all these factors determine inter- and intra-tumour heterogeneity. Both of these types of metabolic heterogeneity are linked to distinct clonal expansion lineages, driven by different sets of oncogenic mutations, and influenced by non-cancer cells in the vicinity. Above all, genetic and metabolic signals are in continuous interaction in the tumour niche and together compose the metabolic output of the tumour. Some examples that highlight the intricacies of a complex genomic–metabolic landscape contributing to the metabolic heterogeneity of tumours are described below.

### Tissue context can influence the effect of driver mutations on metabolism exhibiting inter-tumour heterogeneity

As we have already mentioned, pan-cancer studies have demonstrated that cancers formed from the same tissue or anatomically similar sites show similar metabolic features^2,4^ and identify some metabolic pathways that are unique to certain cancer types, for instance dysregulation of polyamine metabolism in prostate cancers.^1^ Interestingly, both of the studies identified negative correlations when metabolic pathways or overall metabolic gene expression were decreased in cancers in comparison with normal tissues, demonstrating that tumours from metabolically active organs, such as the liver, colon and kidney, lose their specific metabolic functions and shift to a proliferation-supporting programme.^2^ Comparison of metabolomic data from different tumour sets also revealed tissue-specific differences.^11^

Recent studies have demonstrated that the effect of a specific driver lesion on metabolism can be influenced by a tissue context. Differences in glutamine metabolism have been shown for MYC-driven liver and lung tumours,^84^ and branched-chain amino acid metabolism for tumours induced by KRAS activation and TP53 (KP) deletion in lung and pancreas.^85,86^ Evaluating metabolic gene expression patterns in prostate cancer samples also demonstrated that, in contrast, to samples with high MYC levels, samples with high levels of phosphorylated AKT (pAKT) had increased levels of glycolytic genes.^87^ This was consistent with the levels of expression of glucose transporter 1 (GLUT1) in these samples. Importantly, GLUT1 levels were not increased in samples with both high MYC and pAKT levels in comparison with normal prostate tissues, indicating that different genetic drivers can influence the effect of each other on metabolism and thus the outcome. Although KP lung mouse tumours are not sensitive to inhibiting glutamine catabolism,^88^ deleting KEAP1 in these tumours and consequently activating the NRF2 pathway made them heavily reliant on glutamine catabolism,^89^ supporting both glutathione biosynthesis and the activity of the Krebs cycle.^30^ In contrast, the presence of activating mutations in *CTNNB1* increases the expression of its transcriptional target glutamine synthetase (GS), which leads to increased glutamine production and dependence on mTOR pathway activity, regardless of other co-occurring genetic lesions (e.g. ref. ^90^).

This phenomenon has also been observed in human samples. In colon cancer, the expression of *MYC* has been shown to be increased, irrespective of tumour stage, and correlates with the expression of 231 unique metabolic genes,^12^ especially in glycolysis, the pentose phosphate pathway, the purine and pyrimidine biosynthesis pathway, some genes involved in fatty acid biosynthesis and one-carbon metabolism, as well as some metabolite transporters. At the same time, it correlated with the decreased expression of Krebs cycle genes and genes of fatty acid oxidation. In contrast, prostate tumours with high MYC levels did not have increased levels of glycolytic genes although they did have increased levels of the fatty acid biosynthesis pathway in comparison with normal prostate tissues.^87^

### Clonal lineages within the same tissue can contribute to intra-tumour heterogeneity

Breast cancer is an example where different tumour subtypes from the same tissue of origin have different metabolic gene expression patterns, which may be defined by lineage-specific differences. For instance, breast tumours belonging to distinct subtypes exhibit different alterations in glycolysis, gluconeogenesis, tyrosine and retinol metabolism. Basal-like breast tumours show substantial differences in the Krebs cycle, terpenoid backbone and homocysteine biosynthesis, whereas luminal A breast tumours have a unique dysregulation of α-linoleic acid, taurine, hypotaurine, cyclo-oxygenase and arachidonic acid metabolism,^1^ with downregulated nucleotide metabolism.^3^ These differences can be due to changes in the inherent gene regulatory network or genetic lesion composition of basal or luminal cell types or both. Indeed, subtype-specific MMTRs in breast cancer have been identified.^1^

Recent technological advances in genomic studies have allowed the tissue-specific distribution of genetic alteration in tumour suppressors and oncogenes to be clearly demonstrated (reviewed in ref. ^91^). Although for some drivers, tissue specificity is defined by their specific role in a tissue of a tumour origin (i.e. oestrogen receptor *ESR1* in oestrogen-driven cancers; *GATA3* in breast cancer and xeroderma pigmentosum proteins, *ERCC3* and *XPC* in skin), for others the mechanisms of tissue specificity are not fully understood. One of the explanations can be a lineage-specific ability of cells to respond to proliferation signals exerted by specific oncogenes and tumour suppressors defined by their pre-existing epigenetic landscape (reviewed in ref. ^91^).

### Environmental factors that lead to tumour metabolic heterogeneity in both inter- and intra-tumour scenarios

One of the important observations that came out from pan-cancer studies, as we have already mentioned earlier, is that the OxPhos and the Krebs cycle pathways are altered in tumours from different tissues, but there is no conserved signature across tumour types.^1,2,4^ Although tumours in tissues, such as the brain, colon and kidney show a downregulation of OxPhos, tissues including breast and lung have an upregulation of OxPhos.^2^ It may indicate a tissue-specific adaptation of different cancers to the environmental conditions determining a specific tumour type. Indeed, OxPhos expression is negatively correlated with the HIF1α pathway.^2^ Importantly, supporting these results from pan-cancer studies, metabolic analyses of tumour samples from patients infused with ^13^C-labelled glucose demonstrated that while lung and brain tumours had increased glucose catabolism through both glycolysis and the Krebs cycle, clear-cell renal cell carcinomas (ccRCCs) had increased glycolysis but suppressed glucose oxidation through the Krebs cycle.^19^ The effect of environmental factors on intra-tumour metabolic heterogeneity is highlighted in a landmark study of tumour biopsies from metastatic RCC patients.^92^ Combining metabolomics analysis and gene sequencing of different tumour regions with stable isotope labelling of tissue slices revealed regional heterogeneity in pyruvate metabolism. Importantly, sequencing of 23 RCC-associated genes did not show any correlation between mutational status and metabolic clusters.^92^ Furthermore, NSCLC tumours demonstrated intra-tumour heterogeneity of glucose catabolism through glycolysis and the Krebs cycle, which correlated with the degree of perfusion in a given region of tumour tissue as observed by dynamic contrast-enhanced (DCE) magnetic resonance imaging (MRI), but not with the genetic makeup of the whole tumour.^18^ These results demonstrate that environmental factors play an important role in defining the metabolic heterogeneity of tumours.

**Fig. 2:**
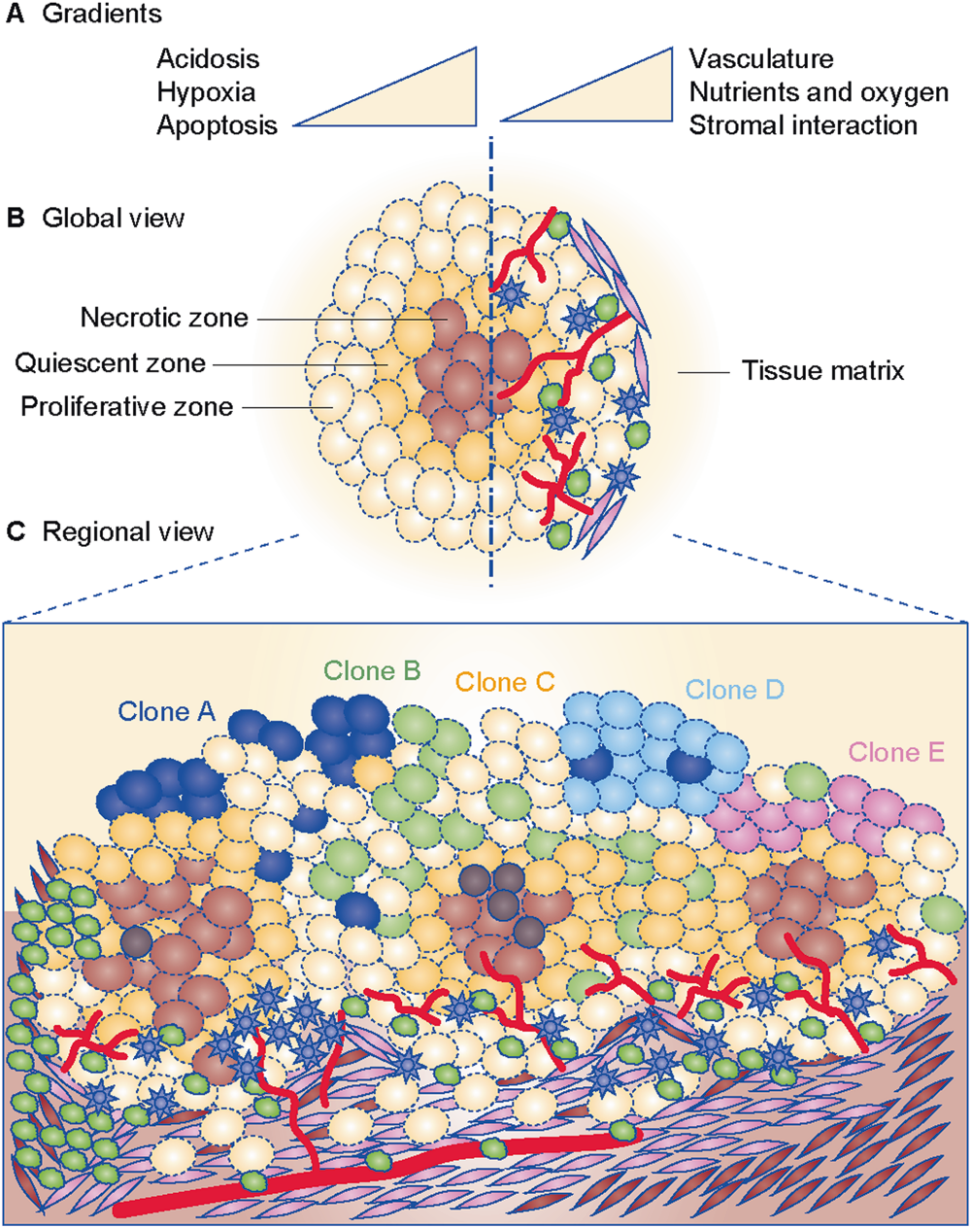
Drivers and contributors of metabolic heterogeneity in cancer. **a** Gradients of regulatory factors in a tumour microenvironment. **b** Global view of a tumour depicting zonation pattern, with the core being most hypoxic, enveloped by a quiescent zone, and margins being proliferative and in continuous interaction with the stroma, vasculature, immune cells (lymphocytes like T cells, B cells and NK cells; monocytes like macrophages or dendritic cells), cancer-associated fibroblasts (CAFs) and host tissue matrix. **c** Regional view of a tumour depicting interaction between multiple zones of hypoxia, vasculature and inflammation, with immune cells in continuous interaction within the tissue matrix. Apart from metabolic factors, genetic clonal heterogeneity is also shown. For panels **b** and **c**, Inflammation is shown by immune cells as represented by lymphocytes (shown as dark irregular green circles) and monocytes (shown as blue stars). Tumour vasculature is shown in red. Clones A, B, C, D and E (shown in navy blue, pastel green, yellow, light blue and pink colour solid circles, respectively) represent multiple cancer cell clonal populations formed by genetic alterations in the same tumour. Cancer stem cells are shown as dark brown circles. Zonation of tumour for Clone C is shown in hues of brown colour, with the darkest zone depicting the most hypoxic and nutrient-starved region. Cells shown with elongated phenotype represent cancer-associated fibroblasts (shown in lavender colour). Stromal cells in tumour microenvironment are shown as brown-coloured elongated cells.

Indeed, the effect of environmental factors contributing to tumour progression have been recently appreciated. Tumour cells interact with immune cells, CAFs and extracellular matrix (ECM), which compose the TME. These interactions are further shaped by fluctuating oxygen and nutrient concentrations due to irregular vascularisation and poor blood supply (see Fig. 2). These interactions are tissue specific and thus would contribute to both inter- and intra-tumour heterogeneity. In a TME, cellular factors (such as immune cells, fibroblasts, vasculature and tumour-associated adipocytes) and non-cellular components (hypoxia and nutrient availability) regulate metabolic reprogramming and considering these factors is absolutely essential in designing efficient therapeutic approaches. Their mode of action and extent of influence is detailed below.

#### Hypoxia

Certain cells within the tumoural mass lack access to blood vessels effectively being cut off from the oxygen and nutrient supply (see Fig. 2). Low oxygen levels induce stabilisation of HIFα proteins, which activate transcription of *GLUT1* and *GLUT3* and glycolytic genes, as well as of pyruvate dehydrogenase kinase 1 (*PDK1*), which in turn deactivates pyruvate dehydrogenase. As a result, glucose catabolism through glycolysis into lactate is increased and its catabolism through the Krebs cycle is decreased.^93^ HIF1α also regulates glutamine uptake through the SNAT2 transporter^94^ and glutamine catabolism through reductive carboxylation, supporting lipid and nucleotide biosynthesis.^95^ Importantly, HIF1α can also accumulate in both hypoxic and normoxic conditions due to the loss of tumour-suppressor functions of *TP53/PTEN/VHL,* as well as activation of the PI3K–AKT–mTOR, MAPK and NF-ĸB pathways (reviewed in ref. ^70^). This suggests that tumour cells with distinct genetic lesions may respond differently to oxygen availability.

#### Nutrient deprivation

Levels of nutrients also play a crucial role in tumorigenesis since it is a process of enhanced cell proliferation and involves enhanced biosynthesis and bioenergetics. In normal cells, AMPK acts as an energy sensor and maintains cells in energy homoeostasis. When nutrients are limited, AMPK increases ATP by promoting the catabolism of glucose and oxidation of lipids, but preventing lipid synthesis and storage.^96,97^ Apart from this, AMPK reduces gluconeogenesis by repressing transcriptional programmes directed via forkhead box O (FOXO) or cAMP-response element-binding protein (CREB).^98,99^ To overcome a tumour-suppressive function of AMPK, transforming cells are under selective pressure to downregulate the pathway. Indeed, inactivating mutations in *LKB1*, a kinase upstream of AMPK activation, are observed in a significant percentage of tumours.^100^ Furthermore, decreased activation of AMPK has been observed in different models downstream of various driver lesions.^101^ However, in already-established tumour cells without *LKB1* mutations, AMPK signalling may assist their survival in nutrient-deprived conditions. Indeed, AMPK has been shown to be required for the survival of melanoma cells downstream of *MYC*, and depletion of AMPK-related kinase 1 (ARK5 or NUAK1) improves survival of mice with *MYC*-induced liver tumours.^102^ Evaluating AMPK activation in mouse tumour models demonstrated its heterogeneous intra-tumour distribution overlapping with hypoxic regions,^103^ supporting its role in promoting cancer cell survival in nutrient-depleted conditions.^104,105^

AMPK plays these distinct roles in regulating metabolic homoeostasis in collaboration with mTOR, which plays a diametrically opposite role to AMPK. mTOR is a serine/threonine kinase that is downstream of the PI3K signalling pathway. Although AMPK can directly target and inhibit mTOR complex 1 (mTORC1), mTORC1 can negatively regulate AMPK in response to leptin signals by inhibitory phosphorylation at certain residues.^106^ Under nutrient-poor conditions, upon ATP depletion AMPK suppresses mTORC1.^107^ Amino acid availability, such as that of leucine and arginine, is a key factor in determining the activity of mTOR and AMPK in conditions of nutrient abundance and starvation in lysosomes via sensors including SLC38A9 (a neutral amino acid transporter), sestrin 1/2 and leucine t-RNA synthetase (leucine sensors) and CASTOR1 (arginine sensor) (reviewed in ref. ^108^). Of these, sestrins promote autophagic catabolism via the AMPK–mTOR pathway and excessive nutrient uptake in cancer cells under stress.^109^ Expression of sestrins is linked to tumorigenesis and is downstream of the TP53, epidermal growth factor receptor (EGFR)–Ras and PI3K–AKT–LKB1 pathways. Since enhanced mTOR activity is a marked feature of tumours, inhibitors like rapamycin^90^ have been shown to be beneficial in some cases, the details of which are discussed in the therapeutic section of this review. Moreover, as described earlier in this review, mTORCs can regulate protein translation, block apoptosis and induce metabolic reprogramming through HIF1α. Hence, the effect of mTOR can be defined by combinations of a genetic lesion in a tumour cell, nutrient status and the environmental context.

#### Immune cells

Both hypoxia and nutrient availability affect metabolism of tumour cells. In addition, metabolism of immune cells is shaped by TME (reviewed in refs. ^110–112^). One of the metabolites that was detected to be consistently elevated in multiple cancer types is kynurenine^11,113^ (see Table 1). Kynurenine is a product of tryptophan catabolism by indoleamine-2,3-dioxygenase (IDO) and it has been demonstrated to have an immunosuppressive function.^114^ Its increased concentration suggests the interaction between tumour metabolism and TME throughout different cancers. Indeed, T cells require glycolysis and glutaminolysis at different stages of growth and differentiation, which is often linked to the expression and activity of the MYC, HIF1α or PI3K–AKT–mTOR pathways (reviewed in ref. ^115^). Furthermore, the nuclear receptor oestrogen-related receptor-α (ERRα) can modulate T-effector cell function by altering the expression of genes involved in glucose metabolism.^116^ Similarly, transcription factor interferon-regulatory factor 4 (IRF4) expression, which is controlled by the strength/affinity of the T-cell receptor (TCR) signal, in turn acts as a regulator of aerobic glycolysis in T-effector cells and is responsible for maintaining their expansion and effector function.^117^ In essence, tweaking the metabolic programming of T cells can drastically alter their functional activity.

Immune cells are heavily reliant on nutrient availability for their activation and function, with glucose being particularly indispensable for cytokine production.^118^ However, it is not just glucose but also other amino acids, such as glutamine and tryptophan, that can modulate their function. A lack of glutamine in cell culture conditions can halt T-cell proliferation and cytokine production.^119^ In addition, it has been postulated that restriction of glutamine in the microenvironment would favour a specific increase in T-regulatory cell populations, which are more immunosuppressive over T-helper or T-effector cell populations.^120^ Conversely, kynurenine suppresses T-effector cell function.^114^ Therefore, by modulating the concentration of metabolites in their environment, tumour cells can affect the metabolism of immune cells. T cells, natural killer (NK) cells or macrophages compete with tumour cells for glucose and other essential nutrients that are required for their function.^121,122^ A subset of dendritic cells (DCs) and myeloid-derived suppressor cells (MDSCs) also participate in this competition. Hypoxia can also induce the expression of PD-1 (programmed death receptor-1) in activated T cells, which, together with nutrient deprivation, contributes to an overall immune-suppressive phenotype (reviewed in refs. ^115,123^). In addition, tumour-associated macrophages (TAMs) differentiate into distinct tumour-promotive populations in a hypoxic and lactate-rich environment. Activation of MAPK signalling and downstream targets such as macrophage-expressing arginase 1 (*ARG1*) and mannose receptor C type 1 (*MRC1*) in TAMs leads to phenotypic and metabolic adaptation.^124,125^ This exemplifies how the anti-tumour activity is impaired in the TME by metabolic insufficiency.

Importantly, the immune cell compartment in a tumour is not only dictated by factors in the microenvironment, but is also influenced by the genetic composition of tumour cells. *KRAS*- and *MYC*-driven mouse lung adenocarcinomas had a marked increase in chemokine (C–C motif) ligand 9 (CCL9) and interleukin-23 (IL-23) inflammatory signalling molecules in the stroma resulting from *MYC* activation.^126^ These molecules facilitate a tumour-promoting microenvironment by supporting angiogenesis, recruiting macrophages and eliminating tumour-suppressive T, B and NK cells. This specific stromal reprogramming could be fully reversed by deactivating *MYC*, which also leads to tumour regression. Oncogenic *KRAS* signalling on its own was also shown to upregulate PD-L1 to create an immune-suppressive environment.^127^

In summary, the genetic and metabolic heterogeneity within tumours and across different tumour types contributes to a strenuous environment for immune cells, determining their composition and function.

#### Cancer-associated fibroblasts (CAFs)

While one major component of the stroma is the immune fraction, the other is CAFs (see Fig. 2). CAFs can be highly heterogeneous since it has recently been shown that they can often originate from fibroblasts, but can also be successors of adipocytes, endothelial cells, bone marrow cells and other epithelial cells. These cells have been shown to contribute to the TME in multiple ways. CAFs originating from bone marrow cells have been shown to provide cysteine to leukaemia cells to resist oxidative stress (reviewed in ref. ^128^). While primary leukaemia cells have low xCt transporters for cystine uptake, CAFs actively import cystine and convert it into cysteine, which can be readily taken up by primary leukaemia cells. This leads to increase in glutathione synthesis, which can confer a protective advantage upon cells from redox stress (reviewed in ref. ^128^). In addition, CAFs in breast and lung cancers harbour activated glycolysis programmes with concomitant increase in lactate production (reviewed in ref. ^128^). Lactate released by CAFs via monocarboxylate transporter 4 (*MCT4*) and taken up by cancer cells via MCT1 activates the TGFβ signalling pathway and enhances mitochondrial activity in cancer cells. This leads to energy synthesis and hence fuelling of cancer cells for extravasation and migration (reviewed in ref. ^128^). Similarly, CAFs from ovarian tumours have increased glutamate–ammonia ligase (GLUL) expression and glutamine production supported from different carbon and nitrogen sources.^129^ Glutamine produced by CAFs is used by cancer cells to support their proliferation and survival in a glutamine-low environment. Finally, CAFs act as a source of proteins for ECM (including collagen, chondroitin sulfate, hyaluronic acid and others) and this is why they have been implicated as barriers in drug efficacy of tumours during treatment.^130^ Together, these factors lead to extensive ECM remodelling, which can promote metastasis and support tumour progression.^128^

The interaction between factors composing the TME can change the fate of specific tumour cells through metabolic reprogramming and drive the selection of more plastic and therapy-resistant clones, further contributing to intra-tumour heterogeneity. Hypoxia and high lactate concentrations have been demonstrated to induce the epithelial-to-mesenchymal transition (EMT) programme that primes cancer cells for a metastatic process.^131^ Transcriptional factors driving EMT can also directly regulate the expression of metabolic enzymes, determining the metabolic profiles of metastasising cells (reviewed in ref. ^132^). Differential metabolic profiles of cancer stem cell (CSC) clones have been suggested to define their ability to metastasise to specific secondary sites (reviewed in ref. ^133^). Moreover, low concentrations of glutamine in a tumour core have also been demonstrated to lead to histone hypermethylation and the subsequent de-differentiation of the tumour cells, fuelling intra-tumour heterogeneity and hence creating therapy resistance.^134^

Therefore, it is clear that tumour metabolism is cumulative of the numerous epigenomic, genomic, transcriptomic, proteomic and metabolic events. Not only it is affected by tissue architecture, but also by the metabolic states of tumour cells and cells composing the TME. The contribution of these factors vastly affects therapeutic success or failure. Furthermore, flexibility of metabolism can represent a significant obstacle in the efficient targeting of metabolic activities in tumours. In the section below, we discuss some of the recent research highlighting the challenges of targeting tumour metabolism.

## Anti-cancer strategies exploiting altered metabolism

Pan-cancer studies have indicated the possibility of identifying metabolic vulnerabilities in a tissue-specific manner. For instance, Rosario et al.^1^ identified a uniquely dysregulated polyamine biosynthesis pathway in prostate cancer samples. Polyamines play vital roles in normal cells, including nucleic acid and chromatin structure maintenance and protein synthesis (reviewed in ref. ^135^). Targeting polyamine metabolism can be quite challenging due to its critical role in cell survival. However, attempts have been made to inhibit the rate-limiting steps of this process, which are regulated by the enzymes ornithine decarboxylase (ODC) and *S*-adenosylmethionine decarboxylase (AdoMetDC) by using competitive binding analogues of their substrates, such as 2-difluoromethylornithine (DFMO) or methylglyoxal bis guanylhydrazone (MGBG) (reviewed in ref. ^135^). The outcome of clinical trials varied from no response to severe cytotoxic effects. Another metabolic feature common among multiple cancer types is increased levels of kynurenine.^11^ As mentioned above, kynurenine is a product of *IDO*, a tryptophan-catabolising enzyme, and is an inhibitor of T-cell proliferation and effector function, and dendritic cell immunogenicity. Although inhibition of *IDO* was initially viewed as a very promising therapeutic target, several clinical trials have failed. Several mechanisms for tumour resistance towards IDO inhibitors have been proposed, including compensation by other tryptophan-catabolising enzymes and channelling of non-catabolised tryptophan into immunosuppressive serotonin and melatonin (reviewed in ref. ^136^). These examples are among the few highlighting the complexity of targeting tumour metabolism.^137,138^ Indeed, (a) the metabolic scenario in tumours is usually complex with multiple factors cross-regulating and interacting in the TME; (b) cancer cell specificity needs to be achieved since these pathways are essential in both normal and cancer cells; (c) efficacy of treatment relies on dosage, which is difficult to gauge due to metabolic flexibility; (d) tumours are metabolically dynamic and can change dependencies post intervention.

Glutamine metabolism as a therapeutic target is one of the examples demonstrating that multiple considerations should be made when designing metabolism-targeting therapeutic approaches. Increased catabolism of glutamine has been observed in various tumours and proliferating normal and cancer cell lines (reviewed in ref. ^139^). CB-839, an inhibitor of glutaminase 1 (GLS1), is in Phase I/II clinical trials (reviewed in ref. ^139^), but unfortunately has limited success. A differential reliance on glutamine catabolism has been demonstrated for tumour models carrying specific genetic lesions,^30,84,89,140^ and this already emphasises the need for better understanding the genomic and metabolic relationship. For instance, Daemen et al.^141^ identified that glutamine is required to support both the activity of the Krebs cycle and glutathione levels to protect cells from ROS. Furthermore, deriving a gene signature of sensitivity towards GLS1 inhibition verified in patient-derived xenograft (PDX) models allowed the authors to predict a higher sensitivity towards inhibiting GLS1 (given a high expression of GAC, a tumour-specific splicing isoform of GLS1) and γ-glutamylcysteine synthetase (an enzyme responsible for glutathione synthesis) in mesenchymal tumours among different cancers (reviewed in ref. ^139^). This prediction is consistent with, for example, the enrichment of the *MYC* transcriptional signature in basal-like/mesenchymal subtype of breast cancers coinciding with increased expression of glutaminase gene and the levels of D-2HG,^8^ and a synthetic lethal interaction between *MYC* and inhibiting glutamine catabolism demonstrated in multiple models.^84,140^ This is also consistent with the reduced capacity of mesenchymal lung cancer cell lines to cope with oxidative stress in response to GLS1 inhibition.^142^ The synergistic effect between GLS1 inhibition and inhibiting glutathione synthesis has also been demonstrated for targeting the proliferation of pancreatic ductal adenocarcinoma (PDAC) cells in vitro and in vivo.^143^

Importantly, investigating the mechanisms of resistance towards CB-839 treatment in cell lines with high expression of *GLS1*, but not sensitive to its inhibition, Daemen et al.^141^ demonstrated that cells resistant to CB-839 were able to upregulate anaeplerotic catabolism of glucose through pyruvate carboxylase. Indeed, one of the important features of metabolism that can significantly contribute to resistance towards metabolism-directed therapies is its extreme flexibility. In addition to the ones mentioned above, other studies have demonstrated the requirement of simultaneously targeting glucose and glutamine catabolism. Combining the EGFR inhibitor erlotinib and CB-839 in *EGFR*-mutant NCSLCs in vitro and in vivo resulted in significantly decreasing glucose and glutamine transport and increasing the AMP/ATP ratio, which resulted in energy crisis, AMPK activation and induction of autophagy.^144^ Targeting mitochondrial catabolism of both glutamine through glutamate dehydrogenase and glucose through the pyruvate monocarboxylate transporter was required to decrease the activity of the Krebs cycle, suppress proliferation and induce death of tumour cells in vitro, and decrease tumour growth in vivo.^145^ Furthermore, aspartate catabolism through the mitochondrial aspartate/glutamate transporter Slc1a3 was demonstrated to support cell survival in the absence of glutamine.^86^ The breakdown of *N*-acetyl-aspartyl-glutamate by expression of glutamate carboxypeptidase II has also been shown to protect high-grade tumours from inhibition of GLS1.^146^

These discoveries demonstrate that targeting more than a single metabolic pathway may be required to sufficiently perturb cancer cell metabolism. This shows how essential it is to understand synthetic lethal combinations based on oncogenic signalling pathways and metabolic pathways in cancer to selectively eradicate cancer cells effectively. Importantly, a few recent studies have demonstrated that metabolism remodelling plays a role in driving the resistance to therapies targeting signalling pathways, and using a combination of inhibitors may be beneficial. For example, treatment with CDK4/6 inhibitors induces the upregulation of MYC protein levels, the subsequent increase in glutamine catabolism and activation of the mTOR pathway.^147^ As a result, CDK4/6 and GLS1 inhibitors demonstrate a synergistic effect in inhibiting proliferation and viability of cancer cells. Inhibition of the mTOR pathway in mouse SCCs induced stabilisation of MYC protein levels through the activity of the glycogen synthase kinase 3a/b (GSK3a/b)–AKT pathway, which was accompanied by increased levels of GLS1.^144^ Inhibiting GSL1 allowed resistance to the mTOR inhibitor to be overcome. Recently, increased glutamine uptake by the SLC38A2 (*SNAT2*) amino acid transporter has been demonstrated to drive hypoxia-mediated resistance to endocrine therapy.^94^ Inhibiting *SNAT2* expression sensitised breast cancer cells to anti-oestrogen treatment.

Several studies have highlighted the differential metabolic requirements of cancer cells in tissue culture conditions versus the host natural environment. For example, Ras-driven lung cancer cells were shown to have varying glutamine dependency in vitro or in vivo based on the abundance of extracellular cystine.^88,148^ Moreover, there are extensive metabolic interactions between tumour cells and factors composing the TME. These interactions should certainly be considered when either designing initial therapeutic protocols or targeting metabolic adaptations arising in response to an initial treatment. For example, since glutamine can be required for both the proliferation of tumour cells and the function of different T cells and proliferation of endothelial cells, GLS1 inhibitors, if targeted specifically to tumour cells, would also increase the glutamine concentration in a tumour milieu. This would make more glutamine available for the anti-tumorigenic cells of the TME. There is also a possible interplay between macrophages and tumour cells that can be shifted by GLS1 inhibitors.^149^ Furthermore, based on the observation that CAFs produce glutamine by GS to fuel glutamine catabolism in ovarian cancer cells, combining inhibiting the expression of *GLUL* in CAFs and *GLS1* in cancer cells was shown to have a profound effect on tumour growth as a whole.^150^ Although multiple transgenic and syngeneic in vivo models with a preserved immune system have been used to evaluate the requirement of metabolic pathways in cancer cells, information on how these perturbations affect the composition of the TME and metabolic activities within it is missing. One of the reasons is the limited capacity of modern techniques to evaluate the activity of metabolic pathways in specific cell populations and regions within tissues in situ. Various imaging modalities such as PET, MRI and in vivo histology using MRI (HMRI) allow non-invasive evaluation of metabolism in patients (reviewed in ref. ^151^). In addition, elegant imaging techniques, including desorption electrospray ionisation (DESI)–mass spectrometry imaging (MSI), rapid evaporative ionisation mass spectrometry (REIMS), matrix-assisted laser desorption ionisation MSI (MALDI-MSI), secondary ion mass spectrometry (SIMS) and imaging CyTOF can be used in semi-quantitative or quantitative modes to identify, analyse and estimate the spatial distribution of metabolites and the activity of metabolic pathways (reviewed in ref. ^152^). Obtaining genetic and metabolic information with spatial co-ordinates using imaging mass spectrometry and cytometry and single-cell sequencing from tumours before and after therapy can provide a holistic view of the genetic and metabolic heterogeneity of tumours, and its dynamics during tumour progression and treatment. Moreover, using predictive modelling, metabolic flux values can be estimated and vulnerable targets can be identified.^153,154^ This is exemplified by identifying glyceraldehyde-3-phosphate dehydrogenase (GAPDH) as a biomarker and a target in excessively glycolytic cells. In this case, GAPDH as a biomarker is independent of the genetic status, but completely dependent on metabolic flux, which can be directly distinguished in the clinic by fluorodeoxyglucose (FDG)–PET.^5,154^ Furthermore, a recent clinical study evaluating the response to metformin treatment in breast cancer patients highlighted the importance of obtaining serial readouts before and after treatment and the integration of metabolic imaging, metabolomics and transcriptomics to identify the heterogeneity of the response that could not be detected at baseline.^9^

## Conclusions

There is growing evidence from oncometabolite, hypoxia and inflammation studies that alterations in metabolism can be drivers of tumorigenesis. This signifies that cancer is a metabolic disease that can be initiated due to genetic or non-genetic signalling or metabolic alterations. In the past, the ease of mapping and measuring genomic and transcriptomic changes in cancer led to tumours being mostly classified based on genomic and transcriptomic signatures, but rarely with metabolic profiles.^1,13,141^ With the upsurge in technological advances in the field of metabolomics in the last decade, it has become easier to understand the precise contribution of deregulated metabolism alongside genomic and transcriptomic alterations. There have been attempts to delineate the effects of genetic and metabolic drivers using multimodal strategies on a cellular basis in situ, but the picture is still far from being clear. The inconsistency and variations in metabolomics studies are evident as described by Reznik et al.,^11^ and the challenges in metabolomics sample processing and data analysis have been recently reviewed by Goveia et al.^155^ Given that metabolic pathways are very flexible and can be remodelled based on a tissue context, tumour architecture and the TME, it is important to integrate their contribution. Furthermore, although there have been significant advances in understanding the regulation and the requirement of metabolic processes in tumour cells and cells composing the TME (reviewed in ref. ^129,156,157^), identifying the dynamic metabolic interactions between different cells in vivo has not yet been achieved. Therefore, it is important to employ multimodal strategies to harness as much information as possible from cancer patients before and after treatment. The knowledge of genetic mutations, altered transcriptional markers and metabolic shifts combined can help identify either synthetic lethal pairs or a combination of drugs against selected targets. Furthermore, the development of novel imaging and analytical approaches is required to be able to detect and visualise the activity of metabolic pathways in vivo with cellular resolution.

## Data Availability

The data used for generating Table 1 are obtained from published studies in open-access journals and are freely available online as supplementary files of the respective articles.
